# Development of a 3D Tumor Spheroid Model from the Patient’s Glioblastoma Cells and Its Study by Metabolic Fluorescence Lifetime Imaging

**DOI:** 10.17691/stm2023.15.2.03

**Published:** 2023-03-29

**Authors:** D.V. Yuzhakova, M.M. Lukina, D.A. Sachkova, G.M. Yusubalieva, V.P. Baklaushev, A.M. Mozherov, V.V. Dudenkova, A.I. Gavrina, K.S. Yashin, M.V. Shirmanova

**Affiliations:** Researcher, Laboratory of Genomics of Adaptive Antitumor Immunity, Research Institute of Experimental Oncology and Biomedical Technologies; Privolzhsky Research Medical University, 10/1 Minin and Pozharsky Square, Nizhny Novgorod, 603005, Russia;; Researcher, Laboratory of Molecular Oncology; Federal Research and Clinical Center of Physical and Chemical Medicine, Federal Medical and Biological Agency, 1a Malaya Pirogovskaya St., Moscow, 119435, Russia; Researcher, Laboratory of Fluorescent Bioimaging; Privolzhsky Research Medical University, 10/1 Minin and Pozharsky Square, Nizhny Novgorod, 603005, Russia;; Master Student, Department of Biophysics; National Research Lobachevsky State University of Nizhni Novgorod, 23 Prospekt Gagarina, Nizhny Novgorod, 603950, Russia; Laboratory Assistant, Laboratory of Fluorescent Bioimaging, Research Institute of Experimental Oncology and Biomedical Technologies; Privolzhsky Research Medical University, 10/1 Minin and Pozharsky Square, Nizhny Novgorod, 603005, Russia;; Senior Researcher, Laboratory of Cell Technologies; Federal Research and Clinical Center, Federal Medical and Biological Agency, 28 Orekhovy Blvd., Moscow, 115682, Russia; Senior Researcher, Laboratory of Molecular Mechanisms of Regeneration and Aging; Engelhardt Institute of Molecular Biology, Russian Academy of Sciences, 32 Vavilova St., Moscow, 119991, Russia; Deputy General Director for Research and Medical Technologies; Federal Research and Clinical Center, Federal Medical and Biological Agency, 28 Orekhovy Blvd., Moscow, 115682, Russia; Head of the Laboratory of Molecular Mechanisms of Regeneration and Aging; Engelhardt Institute of Molecular Biology, Russian Academy of Sciences, 32 Vavilova St., Moscow, 119991, Russia; Junior Researcher, Laboratory of Optical Spectroscopy and Microscopy, Research Institute of Experimental Oncology and Biomedical Technologies; Privolzhsky Research Medical University, 10/1 Minin and Pozharsky Square, Nizhny Novgorod, 603005, Russia;; Researcher, Laboratory of Optical Spectroscopy and Microscopy, Research Institute of Experimental Oncology and Biomedical Technologies; Privolzhsky Research Medical University, 10/1 Minin and Pozharsky Square, Nizhny Novgorod, 603005, Russia;; Junior Researcher, Laboratory of Molecular Biotechnologies, Research Institute of Experimental Oncology and Biomedical Technologies; Privolzhsky Research Medical University, 10/1 Minin and Pozharsky Square, Nizhny Novgorod, 603005, Russia;; Oncologist, Neurosurgeon, Department of Oncology and Neurosurgery, Institute of Traumatology and Orthopedics, University Сlinic; Assistant, Department of Traumatology and Neurosurgery named after M.V. Kolokoltsev; Privolzhsky Research Medical University, 10/1 Minin and Pozharsky Square, Nizhny Novgorod, 603005, Russia;; Deputy Director for Science, Research Institute of Experimental Oncology and Biomedical Technologies; Privolzhsky Research Medical University, 10/1 Minin and Pozharsky Square, Nizhny Novgorod, 603005, Russia;

**Keywords:** tumor spheroids, FLIM, fluorescence lifetime imaging, glioblastoma, metabolic status of tumor cells, NAD(P)H, FAD

## Abstract

Patient-specific *in vitro* tumor models are a promising platform for studying the mechanisms of oncogenesis and personalized selection of drugs. In case of glial brain tumors, development and use of such models is particularly relevant as the effectiveness of such tumor treatment remains extremely unsatisfactory.

**The aim of the study** was to develop a model of a 3D tumor glioblastoma spheroid based on a patient’s surgical material and to study its metabolic characteristics by means of fluorescence lifetime imaging microscopy of metabolic coenzymes.

**Materials and Methods:**

The study was conducted with tumor samples from patients diagnosed with glioblastoma (Grade IV). To create spheroids, primary cultures were isolated from tumor tissue samples; the said cultures were characterized morphologically and immunocytochemically, and then planted into round-bottom ultra low-adhesion plates. The number of cells for planting was chosen empirically. The characteristics of the growth of cell cultures were compared with spheroids from glioblastomas of patients with U373 MG stable line of human glioblastoma. Visualization of autofluorescence of metabolic coenzymes of nicotinamide adenine dinucleotide (phosphate) NAD(P)H and flavin adenine dinucleotide (FAD) in spheroids was performed by means of an LSM 880 laser scanning microscope (Carl Zeiss, Germany) with a FLIM module (Becker & Hickl GmbH, Germany). The autofluorescence decay parameters were studied under normoxic and hypoxic conditions (3.5% О_2_).

**Results:**

An original protocol for 3D glioblastoma spheroids cultivation was developed. Primary glial cultures from surgical material of patients were obtained and characterized. The isolated glioblastoma cells had a spindle-shaped morphology with numerous processes and a pronounced granularity of cytoplasm. All cultures expressed glial fibrillary acidic protein (GFAP). The optimal seeding dose of 2000 cells per well was specified; its application results in formation of spheroids with a dense structure and stable growth during 7 days. The FLIM method helped to establish that spheroid cells from the patient material had a generally similar metabolism to spheroids from the stable line, however, they demonstrated more pronounced metabolic heterogeneity. Cultivation of spheroids under hypoxic conditions revealed a transition to a more glycolytic type of metabolism, which is expressed in an increase in the contribution of the free form of NAD(P)H to fluorescence decay.

**Conclusion:**

The developed model of tumor spheroids from patients’ glioblastomas in combination with the FLIM can serve as a tool to study characteristics of tumor metabolism and develop predictive tests to evaluate the effectiveness of antitumor therapy.

## Introduction

Glioblastoma is registered in 48.3% of all primary malignant brain tumors and is the most aggressive neo-formation [1]. Despite radical treatment tactics, including the maximum possible surgical removal of the tumor, radiation, and chemotherapy, the median survival does not exceed two years.

Current treatment patterns do not take into account individual characteristics of the patient’s tumor, which is a key problem in glioblastoma combatting. Development of a personalized approach to treatment based on the use of tumor models from patient cell material is a promising solution [2-4].

There are two main groups of patient-specific models of glioblastoma: 1) *in vitro* models — monolayer cultures, cultures and co-cultures in scaffolds, 3D spheroids and organoids [5]; 2) *in vivo* models — tumor mice xenografts [6]. At that, there are no unified approaches to obtaining and characterizing such models.

In some tumor models, tumor spheroids have an intermediate position between monolayer cell cultures and animal tumors. Tumor spheroids are three-dimensional multicellular spherical formations of a heterogeneous structure. The morphological heterogeneity of spheroids primarily relates to gradients of nutrients, metabolites, and oxygen. In large spheroids (more than 500 μm in diameter), three main areas can be distinguished: the necrotic core, the inner resting cells area, and the outer actively proliferating layer [7]. It is well known that spheroids express membrane receptors that regulate cell adhesion and metabolism resulting in the intercellular matrix synthesis, which is important for intercellular interaction and interaction with immune cells [8]. On the one hand, tumor spheroids much better imitate all types of intercellular contacts, diffusion of nutrients and oxygen in tumors compared to monolayer cultures and are closer in their characteristics to real tumors. On the other hand, spheroids are much simpler and cheaper to process compared to mouse tumors and organoids, especially in case of brain tumors.

The use of tumor spheroids in preclinical testing of new anticancer agents and therapeutic interventions is of increasing importance [9, 10]. Systems based on spheroids from patient tumor cells are also considered as a promising platform for individual drug selection [11, 12].

In addition to practical problems, patient-specific models, including tumor spheroids, play an important role in the study of tumor metabolism [13]. Significant rearrangements in many metabolic pathways are a tumor characteristic feature, which has been recently considered both as a consequence of malignant transformation and a prerequisite for tumor progression [14]. The most studied features of tumor cell metabolism include increased glycolysis, both anaerobic and aerobic (Warburg effect), impaired mitochondrial respiration, activation of the pentose phosphate pathway, and the use of glutamine and fatty acids as energy substrates [15, 16]. Current studies show that metabolic processes in tumor cells are characterized by high plasticity, whereas tumor cells, even within the same neo-formation, are highly heterogeneous in their metabolic profile, which allows them to quickly adapt and survive in adverse conditions [17, 18]. These aspects of tumor metabolism are less studied.

Metabolic imaging technology based on two-photon fluorescence lifetime imaging microscopy (FLIM, fluorescence lifetime imaging microscopy) is of high potential for studies of tumor metabolism [19-21]. This method is based on estimation of the autofluorescence lifetime of metabolic co-factors such as reduced nicotinamide adenine dinucleotide (phosphate) (NAD(P)H) and oxidized flavin adenine dinucleotide (FAD). These co-factors are involved as electron carriers in many biochemical reactions and are present in the cell in reduced (NAD(P)H, FADH_2_) and oxidized (NAD+/NADP+, FAD) forms. Here, NAD(P)H and FAD exhibit autofluorescence, and at that NAD(P)H typically exhibits a higher fluorescence intensity within tumor cells compared to FAD.

Violation in various energy and anabolic pathways can lead to changes in the autofluorescence decay parameters of metabolic coenzymes, which can be seen at the cell level by means of FLIM [22-24]. Typically, the fluorescence decay of NAD(P)H and FAD is described by a bi-exponential function. In case of NAD(P)H, the short lifetime component corresponds to the free form of the co-factor associated with glycolysis, whereas the long component corresponds to the protein-bound form of NAD(P)H associated with the mitochondrial respiratory chain. For FAD, the short and long components are related to the closed and open conformations of the co-factor, respectively, and the closed one predominates in the protein structure.

**The aim of the study** was to develop a technique to create a patient-specific 3D model of a glioblastoma spheroid and to evaluate the metabolic status of the obtained spheroids by means of FLIM.

## Materials and Methods

### Surgical specimens

All studies on tumor samples were approved by the local ethics committee of the Privolzhsky Research Medical University (Nizhny Novgorod, Russia) (protocol No.12 of August 5, 2022).

Surgical specimens were collected from 5 patients with presumptive (according to preoperative contrast-enhanced MRI) and histologically confirmed diagnosis of Grade IV glioblastoma. Surgery was performed at the Department of Oncology and Neurosurgery of the University clinic of the Privolzhsky Research Medical University. The study was conducted in accordance with the Helsinki Declaration (2013) and approved by the Ethics Committee of the Privolzhsky Research Medical University. Informed consent was obtained from each patient. Tumor samples were approximately of 5×5×5 mm in size. Specimens were transported in ice-cooled test tubes with DMEM (Dulbecco’s modified eagle medium)/F12 (PanEco, Russia) transport medium with a twofold concentration of antimycotic antibiotic (Gibco, USA).

To obtain a primary culture of glioblastoma cells, a tumor tissue piece was mechanically crushed in sterile box in Petri dishes with sterile blades and additionally enzymatically dissociated with the Liberase ΤL reagent (Roche, Switzerland), which is a mixture of type I and type II collagenases, for 5–7 min in CO_2_ incubator (Sheldon Manufacturing Inc., USA). Then, the cell suspension was cleaned from erythrocytes using the lysis ACK buffer (Buffer EL; QIAGEN, Germany). Following centrifugation of the final suspension, the pellet was resuspended in a nutrient medium and transferred into a 25-cm^2^ culture flask or into a 6-well plate (Corning, USA). After 24–48 h, when glial cells adhered to the plastic, the nutrient medium with cell debris was replaced with a fresh complete DMEM with a standard antibiotic concentration.

### Cultivation of cells and spheroids

The cultivated short-term glioblastoma cultures, as well as the U373 MG human glioblastoma stable line, were cultured in a nutrient DMEM containing 10% fetal bovine serum, 0.06% glutamine, 50 U/ml of penicillin and 50 μg/ml of streptomycin sulfate, in CO_2_ incubator at 5% CO_2_, temperature of 37°C and humidity of 85%. Subcultivation was conducted once every 2–3 days when the culture reached the confluency level of 80%. The cells were removed by adding 1 ml of trypsin–EDTA solution (25%) for 5 min. Cell count was conducted in a Goryaev chamber.

To assess cell morphology and viability, the transmitted light images of cells and immunofluorescent images were obtained using a DM IL LED fluorescent inverted microscope (Leica, Germany).

The authors developed an original protocol to create a spheroid model from short-term glioblastoma cell cultures, which is described in detail in the Results section.

Tumor 3D spheroids were formed by culturing tumor cells in 96-well round bottom plates with low adhesion (Corning, USA). To get spheroids from linear U373 MG glioblastoma cells, there were 2000 cells per well seeded. The growth and morphology of tumor spheroids were assessed using a DM IL LED microscope (Leica, Germany). The time points were chosen so as to trace all stages of the tumor spheroid formation: from the beginning of the cell aggregate formation to the cell death. At each time point, 5–7 spheroids were analyzed. Quantitative analysis of images was performed using the ImageJ software (National Institutes of Health, USA), calculating the spheroid area by the following formula: π_^r^_2.

To imitate hypoxia, during day 3 to day 7 of growth tumor spheroids were cultivated in an incubator (Sanyo, Japan) with an oxygen content of 3.5%. The reduced oxygen content was achieved by replacing it with nitrogen. FLIM was performed on day 3 to day 7 of cultivation.

To conduct metabolic imaging, tumor spheroids were transferred to special glass bottom dishes for confocal FluoroDish microscopy (WPI, China) for 2–3 h to attach. An hour before the imaging procedure, the standard nutrient medium was replaced with FluoroLite™ DMEM without phenol red (Gibco, USA).

### Immunocytochemical analysis

Glioblastoma cells were seeded on a flat-bottomed adhesive plastic one day before staining. Cells were fixed with 4% paraformaldehyde solution for 10 min and treated with 0.1% Triton X-100 solution for 5 min; then, they were incubated with primary antibodies to glial fibrillary acidic protein (GFAP) (Boster Biological Technology, Ltd., USA) overnight at the temperature of 4°C, followed by incubation with secondary antibodies conjugated with Alexa Fluor 594 fluorescent label for 1 h at room temperature. The nuclei were counterstained with fluorescent DAPI dye. Imaging was performed using a TX2 filter with excitation of 560/40 nm and emission of 645/75 nm for Alexa Fluor 594 and a CFP filter with 436/20-nm excitation and 480/40-nm emission for DAPI.

### Fluorescence lifetime imaging

FLIM was performed by means of an LSM 880 laser scanning microscope (Carl Zeiss, Germany). A femtosecond Τi:Sa laser (Spectra Physics, USA) with a pulse repetition rate of 80 MHz and a duration of 120 fs was used as the excitation source. Fluorescence lifetime detection was performed using a TCSPC FLIM module (Becker & Hickl GmbH, Germany) based on time-correlated single photon counting. Images were taken with a 40x/1.3 oil immersion objective. NAD(P)H fluorescence was excited in the two-photon mode at a wavelength of 750 nm, the signal was registered within the range of 450– 490 nm. FAD fluorescence was excited at a wavelength of 900 nm, the signal was registered within the range of 500–550 nm. The power of the exciting radiation in both cases was 7 mW. The photon collection time was approximately 60 s. There were at least 5000 photons per pixel. During the experiment, the cells were kept in an incubator at 37°C, 5% CO_2_.

Data obtained by FLIM was processed using the SPCImage software (Becker & Hickl GmbH, Germany). The parameters of the decay curves in each pixel were calculated using the least squares approximation. Fluorescence decay curves for NAD(P)H and FAD were approximated by means of a bi-exponential model. The approximation accuracy was estimated using the parameter ^χ^2. For all data, χ^2^ was within the range from 0.8 to 1.2. The short and long decay components (τ_1_ and τ_2_, respectively), the relative amplitudes (α_1_ and α_2_), as well as the average fluorescence lifetime (τ*_m_*=α_1_τ_1_+α_2_τ_2_) were assessed.

Autofluorescence analysis was performed cell by cell in the area of the cell cytoplasm. For each group, images of 5–7 spheroids were received. In each spheroid, 15– 20 cells were analyzed.

### Statistical analysis

The GraphPad Prism 8.4.3 software (GraphPad Software, USA) was used for comparative data analysis and graphic display. Continuous variables were tested for normality of distribution using the Shapiro–Wilk test (distribution was considered normal at p≥0.05).

During the monitoring of the spheroids growth, the average spheroid area was calculated (for all spheroids in each experimental group on a certain day, 5–7 spheroids in each group). Data is shown as the mean value ± standard error of the mean (M±SEM).

In the FLIM experiments, the average value of the FLIM parameters was calculated using 15–20 cells for each spheroid; then, the average value was determined for all spheroids in each study group (5–7 spheroids in each group).

In a comparative analysis of autofluorescence of NAD(P)H and FAD between spheroids from the U373 MG stable line and the primary culture of glioblastoma, the Student’s t-test was used to find statistically significant differences between the studied groups (differences were considered statistically significant at p≤0.05).

In the hypoxia experiment, the paired Student’s t-test was used to compare indicators in the dependent data groups (differences were considered statistically significant at p≤0.05). The Student’s t-test was also used to find differences between separate data groups (differences were considered statistically significant at p≤0.05).

## Results

### Characteristics of short-term cell cultures

**Figure 1.**
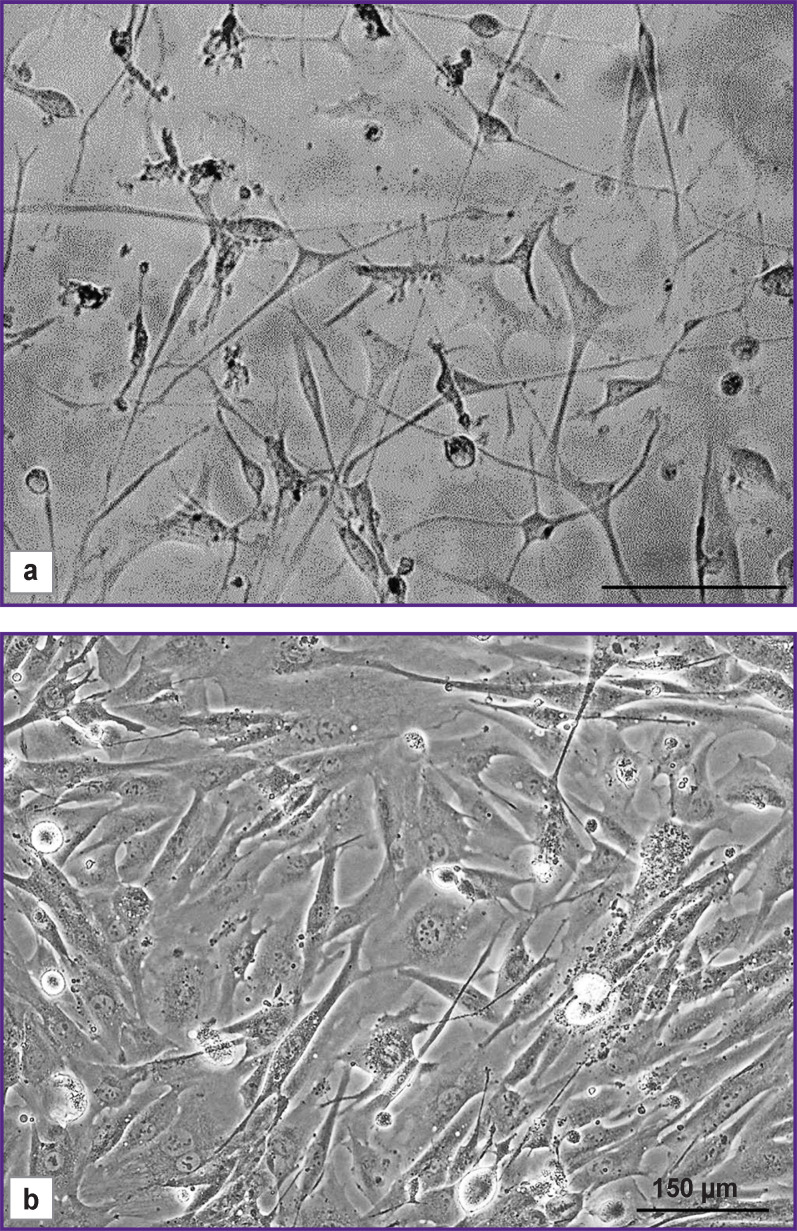
Phase-contrast microscopic images of glioblastoma cells in a monolayer culture Short-term culture of a patient’s glioblastoma with a monolayer density of 40% (a) and 80% (b)

Isolated short-term cultures of glioblastoma cells showed a diffuse distribution of cells and moderate cell polymorphism (Figure 1). Numerous large cells of spindle-shaped morphology (fibroblast-like cells) were registered. Moreover, triangular, oval, round, or irregularly shaped cells were seen. All cells showed pronounced granularity of the cytoplasm and numerous processes.

**Figure 2.**
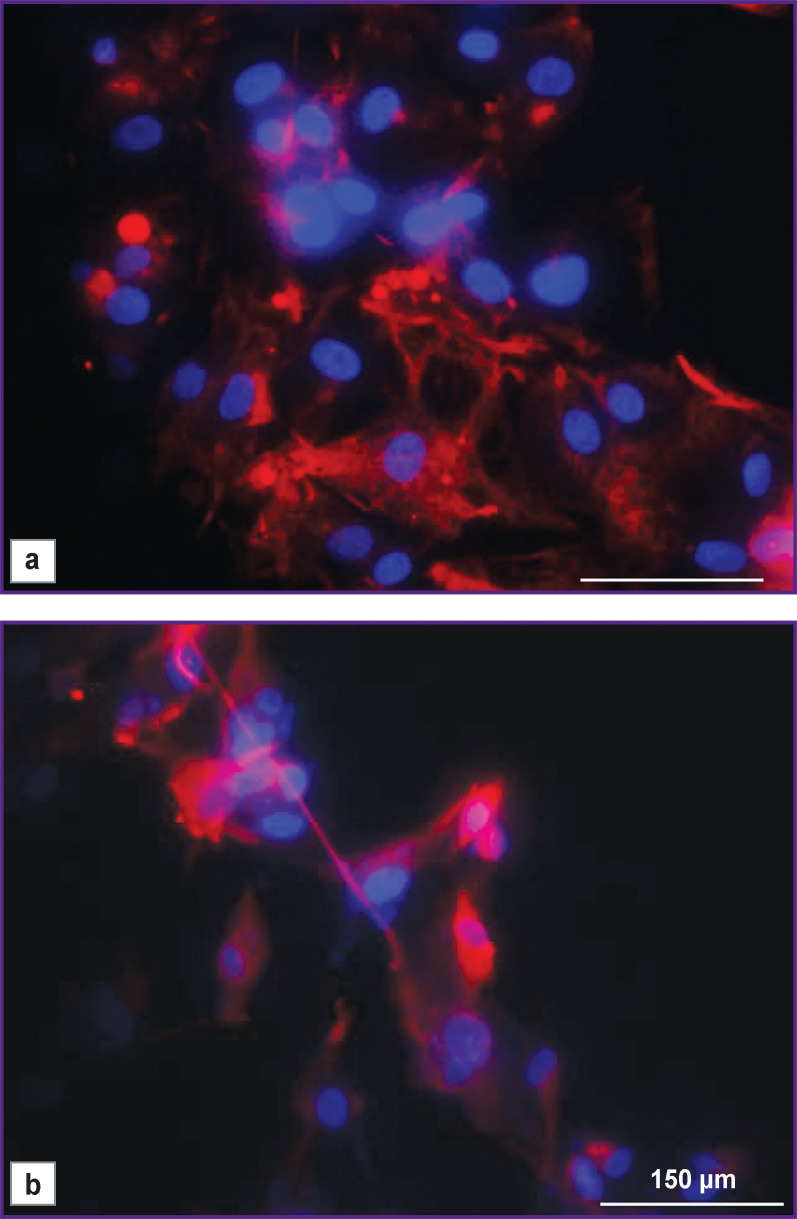
Immunofluorescent microscopic images of glioblastoma cells: (a) a short-term culture of a patient’s glioblastoma; (b) stable line of the U373 MG human glioblastoma. *Red channel —* the GFAP protein in the cell cytoplasm; *blue channel* — cell nuclei stained with DAPI

To confirm the nature of the isolated cells, immunocytochemical staining was conducted for the expression of the main glial marker, the GFAP protein. It was found that all glioblastomas cultures expressed this protein. The human glioblastoma U373 MG stable line, which also expresses GFAP, was used as a positive control (Figure 2).

### Growth and morphology of 3D tumor spheroids cultured from patients’ glioblastoma cells

At the first stage of the study, an original model of a spheroid was developed from a short-term glioblastoma culture.

**Figure 3.**
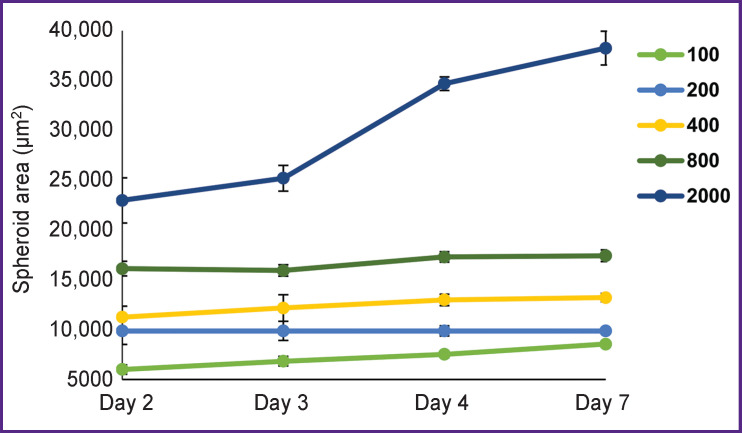
Dynamics of growth of tumor spheroids cultured from a short-term culture of a patient’s glioblastoma The initial concentration of cells with seeding of 100, 200, 400, 800, and 2000 cells per well. Data is shown as the mean value ± standard error of the mean

In order to obtain tumor spheroids from patients’ glioblastoma cells, various numbers of cells for seeding were tested, and their growth dynamics and morphology were compared. It was established that with 100 cells planted in a well a slight growth of the cell agglomerate was observed within 7 days (Figure 3). This agglomerate was of an irregular shape, had a loose structure without a typical optically dense core and a light outer layer, which raised doubts related to formation of a spheroid. With 200, 400, and 800 cells seeded per well, on day 2 of cultivation, non-compact agglomerates of about 40–70 μm in diameter were formed, they did not change during 7 days of growth. It should be noted that with 800 cells seeded per well, the agglomerates acquired a more regular spherical shape. However, spheroids showed almost no growth and development in all indicated concentrations.

**Figure 4.**
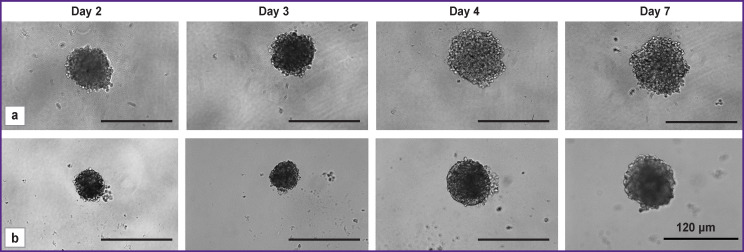
Transmission microscopic images of tumor spheroids from glioblastoma cells obtained on days of growth 2, 3, 4, and 7: (a) a stable line of the U373 MG human glioblastoma; (b) a short-term culture of a patient’s glioblastoma. The initial concentration of cells at seeding is 2000 cells per well

With 2000 cells seeded per well, the spheroids gradually increased in size from 85 to 112 μm and formed a zonal structure during development (Figure 4).

Starting from day 2 of cultivation, cell agglomerates got a correct spherical shape. By day 4 of growth, the spheroids became larger and denser and had a characteristic division into layers: they had a pronounced outer shell with a surface layer of light, actively proliferating cells and a dense dark homogeneous core. On day 7 of growth, mature spheroids began to lose their dense structure, became unconsolidated, which subsequently led to their disaggregation. Based on these results, a concentration of 2000 cells per well was considered optimal to get tumor spheroids from short-term glioblastoma cultures.

When seeded with 2000 cells per well, the U373 MG human glioblastoma stable line demonstrated similar stages of spheroid development, including zoning on day 3 of growth, and a rapid increase in spheroid size from 100 to 134 μm.

The developed technique was reproduced on samples of glioblastomas (Grade IV) from 5 patients. In all cases, the authors observed stable formation of tumor spheroids and their growth with dynamics similar to the presented sample.

### Study of the metabolic status of tumor 3D spheroids using FLIM

#### Comparison of spheroids from patient cells and stable line cells

During the second stage of the study, the metabolic status of tumor spheroids obtained from the patient’s glioblastoma and stable line culture was analyzed. In metabolic imaging tests, spheroids were used on day 4 of cultivation, demonstrating a dense structure with a characteristic division into zones.

Autofluorescence images of NAD(P)H and FAD coenzymes in spheroid cells were obtained by means of FLIM, and the lifetime parameters were analyzed. In the case of NAD(P)H, the lifetime of the free (τ_1_) and protein-bound forms (τ_2_) varied within the range from 0.33 to 0.41 ns and from 2.46 to 2.63 ns, respectively, and did not differ significantly for tumor cell cultures. At that, for FAD, the lifetimes of the closed (τ_1_) and open (τ_2_) conformations differed depending on the cell culture and were statistically significantly longer in spheroids formed from patient cells compared to the stable line (see the Table). Here, the absolute values of the fluorescence lifetimes of the NAD(P)H and FAD coenzymes recorded in all glial cells corresponded to the typical values reported in the literature [19, 23].

**Table:** Fluorescence lifetime parameters of NAD(P)H and FAD coenzymes in tumor spheroid cells (M±SEM)

Coenzymes	Lifetime (ns)	U373 MG line cells	Short-term glioblastoma culture (Grade IV)
NAD(P)H	τ*_m_*	0.79±0.36	0.77±0.41
	τ_1_	0.36±0.14	0.37±0.40
	τ_2_	2.60±0.51	2.55±0.80
FAD	τ*_m_*	0.77±0.22	0.94±0.72*
	τ_1_	0.38±0.19	0.46±0.37*
	τ_2_	2.63±1.04	3.05±1.81*

* statistically significant differences for spheroids from the U373 MG line cells, p≤0.001.

**Figure 5.**
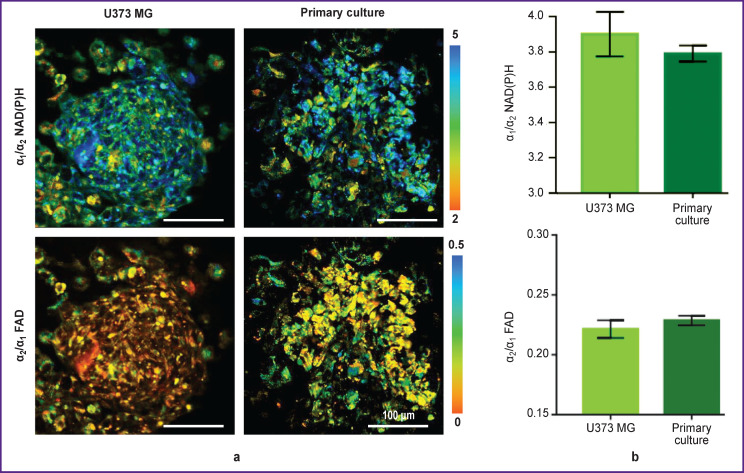
Study of the metabolic status of glioma cells in the 3D tumor spheroid model: (a) microscopic FLIM images of the ratio of α_1_/α_2_ NAD(P)H and α_2_/α_1_ FAD contributions in spheroid cells; images were taken at a depth of 20–30 μm; (b) quantitative assessment of α_1_/α_2_ NAD(P)H and α_2_/α_1_ FAD in spheroid cells. Bar charts reflect the mean value ± standard error of the mean

The ratio of the free NAD(P)H to the bound protein (α_1_/α_2_) contributions in spheroids obtained from the primary glioblastoma culture (~4.6) and the U373 MG stable line (~4.8) was similar (Figure 5). The ratios of FAD contributions in the open and closed conformations (α_2_/α_1_) also were not statistically significantly different in spheroids from patients’ glioblastoma (~0.22) and line culture U373 MG (~0.22).

**Figure 6.**
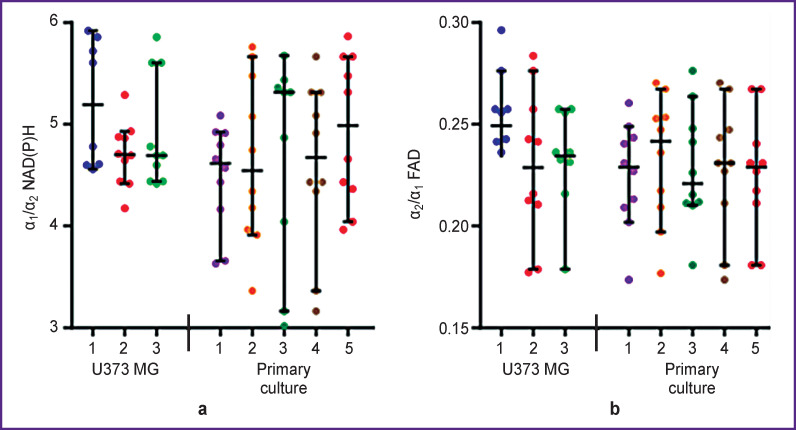
Metabolic heterogeneity of tumor spheroids at the cell level: (a) quantitative assessment of the ratio of free NAD(P)H to protein-bound NAD(P)H contribution according to FLIM images, α_1_/α_2_; (b) quantitative assessment of the ratio of the open and closed FAD conformations contributions according to FLIM images, α_2_/α_1_. The scatter plots show the measurements for individual cells inside spheroids, and the horizontal and vertical lines provide the mean value ± standard deviation. Numbering within the groups corresponds to individual spheroids obtained from the same cell culture

Based on the FLIM, NAD(P)H, and FAD data, the intercellular heterogeneity of metabolism in each spheroid was analyzed in more details. Comparison of optical metabolic parameters for the peripheral and central zones of spheroids did not show metabolic zoning. The mean values of the α_1_/α_2_ NAD(P)H and α_2_/α_1_ FAD ratios in spheroids from the same culture differed insignificantly. At that, intercellular heterogeneity of metabolism in each spheroid was higher in case of spheroids obtained from primary glioblastoma cultures compared to spheroids from the U373 MG line culture, which was expressed in a large spread of values of α_1_/α_2_ NAD(P)H and α_2_/α_1_ FAD (Figure 6).

#### Changes in metabolism under hypoxia conditions

The oxygen status of malignant neoplasms is currently considered as one of the key factors determining the disease prognosis and the therapy effectiveness [3].

In order to establish whether the autofluorescence parameters of spheroids are sensitive to metabolic rearrangements induced by hypoxia, we used the FLIM method to study tumor spheroids formed from short-term cultures of glioblastomas under normoxia (21% O_2_) and hypoxia (3.5% O_2_).

**Figure 7.**
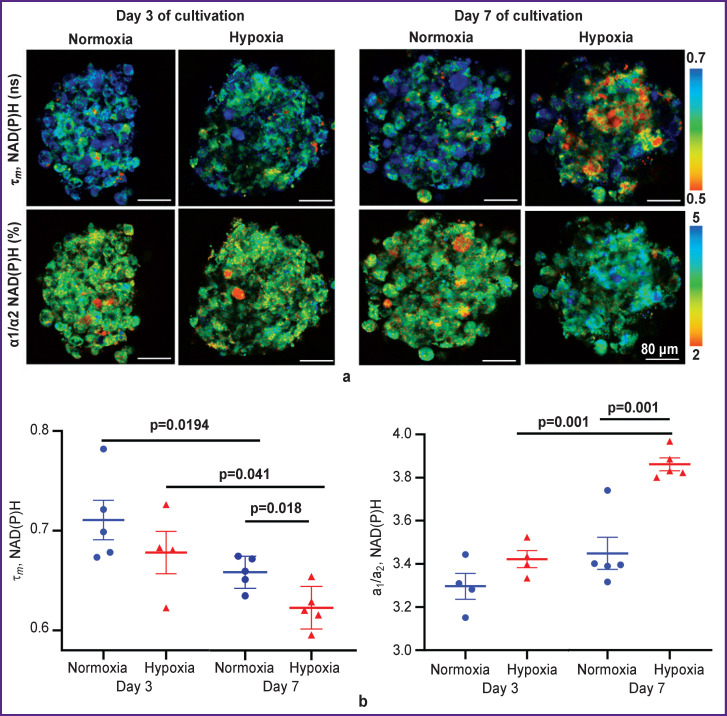
FLIM images of 3D spheroids from a patient glioblastoma: (a) representative FLIM images of spheroids in the NAD(P)H channel; (b) quantitative analysis of the mean lifetime τ*m* and the ratio of the free NAD(P)H to protein-bound NAD(P)H α_1_/α_2_. The scatter plots show measurements for individual spheroids, and the horizontal lines demonstrate the mean value ± standard error of the mean

It was established that starting from the day 3 of cultivation of spheroids under hypoxia, there was a trend to a decrease in the average lifetime τ*_m_* of NAD(P)H (from 0.71±0.02 to 0.68±0.02 ns) due to a decrease in the contribution of the bound component α_2_ (from 23.11±0.30 to 22.62±0.20%), in contrast to normoxia (Figure 7 (b)). On day 7 of cultivation, this trend became more pronounced. The decrease in τ*_m_* and α_2_ was statistically significant: 0.62±0.01 ns and 20.60±0.10% under hypoxia compared to 0.65±0.01 ns and 22.50±0.40% under normoxia (p=0.018 and p=0.001, respectively). The observed changes were expected and were associated with the cell transition to a more glycolytic metabolism under decreased oxygen content conditions.

One should note that in more mature spheroids (day 7 of growth), the values of τ*_m_* and α_2_ NAD(P)H were lower than in “younger” spheroids (day 3), both under normoxia and under hypoxia, which may indicate a shift in metabolism towards glycolysis. The increase in glycolysis with time during the natural growth of the spheroid may be due to the limited diffusion of oxygen into spheroids with a dense structure.

Therefore, the study conducted with the FLIM method showed that tumor spheroids from the primary glioblastoma culture obtained according to our protocol were similar in their metabolic features to spheroids from the standard cell line, adequately responded to changes in the oxygen content in the microenvironment, and demonstrated a higher degree of intercellular metabolic heterogeneity.

## Discussion

Glioblastomas are known for their unique tumor microenvironment, hypoxia, high heterogeneity in the neo-formation, and significant differences between tumors in different patients. This is the reason for turning from the use of the “established” transformed glioma lines and monolayer cultures to the use of patient-specific models that are close in structure to a solid tumor when studying tumors [25, 26].

Short-term cell cultures obtained from the patient surgical specimen retain the molecular profile and cell heterogeneity of the original tumor, thus reflecting the biological characteristics of tumors better than commercial cell lines [27].

The receipt of a tumor cell culture from a patients’ tumor is difficult due to frequent contamination of the primary material and rapid growth of stromal cells. The literature sources provide several methods for obtaining temporary tumor cultures, the major methods include direct culturing of tumor tissues (pieces or cell suspensions) and using of xenografts, where the animal organism acts as the primary recipient of tumor cells [28, 29]. A significant disadvantage of the latter method is the undesirable selection of tumor cells in the animal body. In this regard, the method of direct decultivation of tumor cells is considered more relevant. The culture tissue should have no necrotic areas, be as sterile as possible and sufficiently rich in the cells to be cultured. Unlike many other types of tumors, gliomas are characterized by a good yield of viable tumor cells. Hence, in case of glial tumors, especially glioblastomas, the probability of getting primary cultures is the highest compared to other tumor localizations.

As a result of the study, we developed a 3D model of a tumor spheroid based on a short-term culture obtained from the patient surgical specimen, and compared its characteristics with the characteristics of spheroids from a glioblastoma line cell culture. In literature, spheroids are mainly obtained from surgical samples of a patient’s tumor, specially processed tumor pieces with a diameter of 200– 400 μm, which are placed in agar with a nutrient medium [30], or after sample fermentation, the cell suspension is immediately placed on plastic with a low-adhesion bottom, where spheroids are formed spontaneously [31]. To set a standard for the growth characteristics of spheroids in this study, we used the method of growing spheroids on round-bottom low-adhesion plates from a primary cell culture with a fixed number of cells (2000 cells in 200 μl of medium per well) for seeding, which provided for getting dense multicellular structures with reproducible growth during 7 days.

In case of using line tumor cell cultures, the number of cells seeded in a well to obtain spheroids usually ranges from 100 to 5000 cells, depending on the rate of their proliferation [32-34].

During the study, we analyzed metabolic parameters in tumor spheroids using the FLIM method of endogenous coenzymes NAD(P)H and FAD. Due to its non-invasiveness and no need to introduce exogenous dyes into a cell or tissue, this method is a powerful tool to assess the metabolic status at the cell level and proved itself in studies of tumor metabolism *in vitro* and *in vivo* [20-24]. However, there are few studies using the FLIM method on tumor spheroids or similar structures. We have earlier studied the metabolic status of tumor spheroid cells obtained from the stable line of human cervical cancer HeLa using FLIM [33], and for the first time demonstrated heterogeneity of cell metabolism due to different proliferative activity of cells at the periphery and in the center of the spheroid. It was found that the cells of the spheroid periphery (zone of active proliferation) had a more glycolytic type of metabolism compared to the cells of the central area (rest zone). The current study lacks metabolic zonation in tumor spheroids from glioma cells, which can be explained by other ways of their growth, for example, a more moderate growth rate or a diffuse distribution of proliferating cells.

In a number of studies, the FLIM method was used to assess the metabolic parameters of tumor cell organs, that are multicellular 3D structures from the patient tumor specimen containing various cell types [13, 35, 36]. In their recent study, Morelli et al. [13] suggested a cell organ model from patients’ glioblastoma cells in combination with FLIM to predict response to temozolomide treatment. It should be noted that, unlike spheroids, cell organ size and composition are uncontrolled, thus their handling requires more data collection and complicates interpretation of the results. According to our information, there were no studies on the metabolic status of glial spheroids based on primary material using the FLIM method conducted, which determines the relevance and novelty of the study.

The performed study showed that tumor spheroids from patients’ glioblastomas practically do not differ in optical metabolic parameters from glioblastoma stable line spheroids and adequately respond to hypoxia with a change of the metabolic profile to a more glycolytic profile. In case of hypoxia, spheroids demonstrated a decrease in the relative contribution of the bound form of NAD(P)H associated with oxidative phosphorylation. Chronic hypoxia is a typical physiological feature of many solid tumors, including glioblastomas [37]. Hypoxia indirectly affects signal transduction pathways and regulation of several genes and proteins transcription, thus being an independent factor for tumor progression [38]. In glioblastomas, hypoxia correlates with resistance to radiation and chemotherapy. It is important to observe metabolic rearrangements under hypoxic conditions during natural growth and therapeutic effects on glioblastoma cells in an *in vitro* model to understand mechanisms of adaptation and development of tumor resistance.

Fluorescence lifetime imaging of metabolic co-factors is a highly sensitive tool for assessment of the early response of tumor cells to chemotherapy. As well as other researchers [20, 23, 24, 33, 39], we demonstrated this in various tumor models *in vitro* and *in vivo*. The FLIM method is a promising tool for prediction of the drug therapy effectiveness for patients by assessing the metabolic parameters of cells isolated from a tumor after drug exposure *in vitro*. The tumor model created in this study is based on patients’ glioblastoma cells and the FLIM metabolic imaging technique may become a methodological basis for development of personalized approaches in glioma treatment.

## Conclusion

The 3D model of tumor spheroids created *in vitro* from cells isolated from the surgical specimen of patients’ glioblastomas allowed to get dense spheroids with stable growth, demonstrating a high degree of cell metabolic heterogeneity and an adequate response to hypoxia. The developed model of tumor spheroids from patients’ glioblastomas in combination with the FLIM method can become a tool for studying specifics of tumor metabolism, predicting the effectiveness of antitumor therapy for patients, and preclinical testing of new drugs.
